# Vaccine Hesitancy in Japan: From a Perspective on Medical Uncertainty and Trans-Scientific Theory

**DOI:** 10.12688/f1000research.125159.2

**Published:** 2023-01-11

**Authors:** Miwako Hosoda

**Affiliations:** 1 Faculty of Life Network Science, Seisa University, Yokohama, Japan

**Keywords:** Vaccine Hesitancy, Japan, HPV vaccine, Uncertainty, Trans-Science

## Abstract

The development and dissemination of vaccines has made immunization possible and has led to the successful control and eradication of various infectious diseases in many parts of the world. However, even when vaccines that are said to be "effective" are offered, a certain number of people do not receive them, and this has become a problem known as "vaccine hesitancy”. ItThe reason of “Vaccine hesitancy” is pointed out that there is not only because of the risk of contracting infectious diseases if they are not vaccinated, but also because of the lack of a collective immunity system. Vaccines are an effective means of acquiring immunity, but no matter how highly effective vaccines are developed, if the vaccination rate does not exceed a certain number, “herd immunity”, which means that the risk of person-to-person transmission is reduced when a significant portion of the population becomes immune to, cannot be acquired. Therefore, how to increase the vaccination rate of the population is a major public health challenge. This paper reviews previous studies on "vaccine hesitancy" in Japan and analyzes people's hesitancy in terms of negative "rumors" about vaccines, risk perception of vaccine side effects, and sense of burden when receiving vaccinations. Then, the author will examine that the background of "vaccine hesitancy" is not only distrust of vaccines and risk perception of side effects, but also distrust of those who provide and promote vaccinations, such as medical professionals, government, and public administration. By using medical uncertainty which shows there are many unknowns and uncertainties in medicine and trans-scientific theory which indicates there are areas that science cannot answer this paper argues that the problem of "vaccine hesitancy" can be reduced if medical professionals and governments show sincere empathy and attitude toward victims of adverse vaccine reactions and those who hesitate to vaccinate.

## What is vaccine hesitancy?

The development and widespread use of vaccines has drastically changed the situation in which the only way to prevent infectious diseases was through behavioral changes, such as quarantine. Vaccines have made immunization possible and have been successful in controlling and eradicating various infectious diseases in many parts of the world. However, even when a vaccine that is “scientifically” effective according to peer-reviewed academic papers is offered, a certain number of people do not take the vaccination, and this has become a problem known as “vaccine hesitancy.” It is estimated that 5–10% of the vaccine hesitant population has strong antipathy toward vaccines. It is also clear that there is a significant percentage of people who hesitate to vaccinate even if they do not have such clear intentions. ^1^^,^^2^

Why is “vaccine hesitancy” a problem? It has been pointed out that the reason is not only because of the risk of contracting an infectious disease if they are not vaccinated, but also because of the lack of a “herd immunity” system. “Herd immunity” means that by immunizing everyone, it is possible to lower the risk of infection for those in the group who cannot be immunized ( *e.g.*, those who are highly susceptible to certain pathogens or allergic). ^3^ The presence of a large number of people in a given population who are immunized against an infectious disease through vaccination reduces the infection rate in the population.

Vaccines are an effective means of acquiring immunity. However, no matter how highly effective a vaccine is developed, if the vaccination rate does not exceed a certain number, “herd immunity” cannot be acquired. “Herd immunity” means that the risk of person-to-person transmission is reduced when a significant portion of the population becomes immune to an infectious disease. While it is not appropriate to overemphasize the benefits of “herd immunity”, it is certainly possible to confirm its substantial effectiveness. ^4^^–^^8^ Therefore, it is said that how to increase the vaccination rate of a population is a major public health challenge. Therefore, the fact that many people do not receive vaccinations due to “vaccine hesitancy” is problematic.

How, then, does this “vaccine hesitancy” arise? To preemptively conclude, “vaccine hesitancy” is not only caused by distrust of vaccines or the risk of vaccine side effects, as is generally believed, but rather by distrust of the entities that promote vaccination. In other words, people’s distrust of the medical profession, government, and administration is thought to be the cause of their “vaccine hesitancy.” In fact, no matter how much the medical profession, government, and administration scientifically prove the safety of vaccines, and no matter how much they claim that people’s health damage is not caused solely by vaccine side effects, it may change the mindset of those who are at some vaccine hesitant, but it cannot reduce it to zero. Various approaches have been pointed out to reduce vaccine hesitancy, including education, health literacy, and consideration about individual stress level. ^9^^–^^13^ The aforementioned “vaccine hesitant” people, those who hesitate to vaccinate even if they do not have such clear intentions, do not have a sense of trust in the camps that recommend vaccines: vaccine manufacturers, the medical profession, the Ministry of Health, Labor, and Welfare, and politicians.

This paper will begin with a review of previous studies in public health and in medicine and health sciences on “vaccine hesitancy” in Japan. By doing so, the author will confirm that most studies analyze people’s hesitancy toward vaccines in terms of negative “rumors” about vaccines, risk perception of vaccine side effects, and sense of burden when receiving vaccinations. ^14^^,^^15^ Then, by using literature and news reports of patient’s narrative, the author points out that the background of “vaccine hesitancy” is not only distrust of vaccines and risk awareness, but also distrust of those who provide vaccines and promote vaccines, such as medical professionals and the government. Finally, the author suggests that the problem of “vaccine hesitancy” can be reduced if medical professionals and governments show sincere empathy and attitude toward vaccine side effect victims and those who are hesitant to vaccinate.

## Overview of immunization in Japan

It is said that vaccine-preventable diseases are best prevented by vaccination. In the past, there was a persistent “rumor” that the measles, mumps, and rubella combined (MMR) vaccine could cause autism. And even now that this has been scientifically proven to be false, “vaccine hesitancy” remains. ^16^ And some parents make the decision not to have their children receive MMR vaccinations. Japan is no exception, and this skepticism about vaccines existed in the past and still exists today.

The HPV vaccine is intended to prevent cervical cancer, but the HPV vaccination has been a great problem in Japan over the years. In Japan, public subsidies for the HPV vaccine began in 2011, and in 2013, the vaccine became a routine vaccination administered mainly by municipalities based on the law. At that time, girls between the ages of 13 and 16 years were eligible for the public subsidy. According to The Japan Society of Gynecologic Oncology, the HPV vaccination rate was as high as approximately 70% when this system was first introduced. ^17^^,^^18^

However, adverse events which were called “a variety of symptoms” occurred in girls after HPV vaccination. These symptoms included generalized cramping and the inability to stand or walk after vaccination. These symptoms of girls were repeatedly reported by the mass media on TV and in newspapers as side effects of the vaccine. ^19^ In addition, a great deal of information about the side effects of HPV vaccine was also circulated on social networking services, further increasing the public’s distrust of the vaccine.

As a result, many girls and their families became hesitant to receive the HPV vaccine. In June 2013, the Ministry of Health, Labor, and Welfare (MHLW) announced that it would no longer actively recommend the HPV vaccine. The MHLW stated, “We will not exclude it from the routine vaccination program. However, it has decided not to actively recommend the vaccine. This was then announced to the general public. The specific wording of the MHLW’s statement at that time was, “We do not actively recommend the vaccination,” and “Please take the vaccination after understanding its benefits and risks.

This decision by the MHLW led the public to recognize that the government had acknowledged the dangers of the HPV vaccine. Since then, the HPV vaccination rate has plummeted to less than about 1% in just over three years. This withholding has continued for a long time, and by 2020 the vaccination rate had dropped to 0.1%. ^20^ In Europe and the USA, the HPV vaccination rate is between 70% and 80%, so Japan’s low vaccination rate is outstanding.

It was under these circumstances that the COVID-19 pandemic occurred. Initially, there was no vaccine against COVID-19, and infection was prevented through behavioral measures such as maintaining social distance and wearing masks. However, a vaccine was developed at the end of 2020, making prevention possible. In Japan, a system was prepared for gradual intake starting around the beginning of 2021. The COVID-19 vaccine has been accepted by a relatively large number of the population, with nearly 80% of the population vaccinated for the first and second doses; the third dose is lower, at about 60%, but not lower than in other countries. ^21^

At the same time, however, many negative “rumors” circulated about the current COVID-19 vaccine, which is a newly developed mRNA-based vaccine. And many people chose not to receive the vaccine. The following is an overview of the reasons for vaccine hesitancy based on previous studies on such “vaccine hesitancy.”

## Hesitations about vaccines 1): negative “rumors” about side effects and vaccination itself

The media reports of serious side effects, and the spread of such rumors on social networking sites are commonly cited as major reasons for people’s hesitation to get vaccines. ^22^ For example, with regard to the HPV vaccine, “rumors” ran rampant, ranging from “the side effects are so severe that it makes it impossible to lead a daily life,” to “getting the vaccine makes you infertile,” to “the HPV vaccine is a government conspiracy”.

The following “rumors” about COVID-19 vaccine also spread on SNS: “vaccinations do not work”, “side effects are scary”, “vaccines weaken the human immune system”, and “young women will not be able to have children if they are vaccinated”. Such “rumors” are flying around, and even the opinion that “vaccination is not necessary because antibodies will be produced if the infection occurs naturally” is being circulated. This situation is often evaluated as “low health literacy”. ^14^

However, it is also true that some people have suffered serious health problems due to vaccine-induced side effects. For example, even for the COVID-19 vaccine, the MHLW has so far accepted 3,680 applications for relief under the Immunization Health Damage Relief Program. ^23^^,^^24^ And 850 people have been identified as victims of adverse reactions to coronavirus vaccines through the examination. Among them, a woman in her 90s who developed acute allergic reactions and other symptoms after receiving a new coronavirus vaccine and died was included. Thus, it is true that there are health hazards, including deaths, caused by vaccination, so it is necessary to avoid assuming that those who hesitate to be vaccinated are simply influenced by “rumors” in general.

## Hesitancy toward vaccines 2): risk perception toward vaccines

Vaccines come with the risk of side effects, and adverse reactions can occur after vaccination. Immunization has the advantage of preventing diseases (benefit), but also has adverse reactions and serious side effects (risk). The Immunization Law in Japan provides for the prevention of infection through the safe administration of vaccinations, as well as for remedial measures in the event of risk. The vaccination system is designed to address unavoidable risks.

People with underlying diseases or immune disorders, such as allergies, are considered to be at high risk from vaccinations. Thus, for these people, the risks are higher than the benefits of receiving vaccinations. So, it is precisely because there are potentially people in society who cannot be immunized that it is necessary for those who can be immunized to be vaccinated to prevent infectious diseases. This is the concept of “herd immunity,” which is a basic public health mechanism. ^25^

However, if one takes the view that everyone is at risk for vaccination, it becomes difficult to achieve a form of “herd immunity.” This would increase the risk of infection for those who are constitutionally unable to receive vaccinations. Because people have corrected information that vaccinations cannot avoid side effects, they may weigh the benefits of vaccinations against the risks and decide that the risks are greater. Some people try to avoid the risks even when experts agree that the benefits are greater and the risks are smaller. In such cases, it would not be possible to say that such people have low health literacy.

## Vaccine hesitancy 3): burden to vaccinate

Rumors and concerns about risks are not the only reasons for vaccine hesitancy. Several studies have shown that the burden of time and cost are the main reasons for “vaccine hesitancy.” For example, there are many types of vaccinations for pediatric infectious diseases. While other countries offer a combination of six vaccines, Japan does not offer simultaneous vaccination with the exception of the MR vaccine. For this reason, parents have to adjust their own schedules and take their children to medical institutions for multiple vaccinations. It has been reported that this high burden for parents leads to “vaccine hesitancy”. ^26^

The first of these burdens is the time burden. ^27^ In order to receive the vaccine, children must be taken to hospitals and clinics during the daytime when they are open. However, many parents find it difficult to find the time to do so. Often, dual-workers or single-parent families limit the time of day when they can take their children to a medical facility. If they have more than one child, they must ask someone else to babysit, and taking time off work is not an easy task. Second, the vaccine schedule is complicated and burdensome to keep track of. ^28^ However, in Japan, where the practice of having a family doctor is not common, parents are basically responsible for setting up their own vaccine schedule and many parents find this difficult. Third, the number of medical institutions where pediatricians work has been decreasing. In 2008, there were 2906 pediatric facilities, but by 2018, the number had decreased to 2567. ^29^ The resulting decrease in the number of places where vaccinations can be given has also contributed to the difficulties felt by parents. The need for pediatric clinics that are open on weekends has also been pointed out so that parents with weekday jobs can see their children even on weekends. ^30^ Furthermore, many parents feel a financial burden as a fourth factor. The vaccinations themselves are free of charge, as they are covered by public funds. However, the cost of transportation from home to the medical institution, the cost of babysitting if there are other children, and the loss of income due to absence from work are cited as financial burdens. When parents are low-income status, the vaccination rate of their children is markedly lower. ^31^

Such circumstances that increase the financial burden reflect the current state of Japanese society, in which parents work long hours due to their unstable work style of part-time work and reduced income caused by the recession and divorce. In addition, family members’ circumstances, such as divorce, family discord, and the lack of other reliable guardians due to non-Japanese nationality, are also considered to be causes of “vaccine hesitancy.”

## Changing treatment of vaccines

As we have seen above, negative “rumors” about vaccines, concerns about the risk of side effects, and the financial and time burden of vaccinating have been pointed out as reasons for “vaccine hesitancy.” This is also true to some extent for the COVID-19 vaccine. In a study that examined the percentage of Japanese hesitant to receive the COVID-19 vaccine and investigated factors associated with “vaccine hesitancy,” 11.3% (10.9–11.7%) of the 23,142 responses analyzed were hesitant to receive the COVID-19 vaccine. ^32^ This breakdown of “vaccine hesitancy” was higher among young adults and women, particularly among young women (15.6%). On the other hand, the percentage of “vaccine hesitancy” was the lowest among older men, at 4.8%. The most frequently cited reason for not vaccinating was fear of adverse reactions to the vaccine. More than 70% of respondents were concerned about adverse reactions to the vaccine. This percentage of “vaccine hesitancy” toward COVID-19 in Japan was comparable to that of previous studies overseas.

The age range for COVID-19 vaccination has been gradually lowered in Japan. In conjunction with this, some studies have evaluated factors related to parents’ “vaccine hesitancy” toward COVID-19. An online survey of parents of children aged 3–14 years living in Japan revealed that parents’ “vaccine hesitancy” toward COVID-19 was correlated with the way they obtained information about the vaccine. ^33^ That is, those who cited social media as the most reliable source of information were found to be more hesitant about vaccination than those who trusted official information. Furthermore, more mothers than fathers, and more people with low than high awareness of infection risk, felt “vaccine hesitancy”. The study also showed that those with lower levels of satisfaction with social relationships were more likely to be hesitant to vaccinate.

In summary, those who are more concerned about the risk of adverse reactions to vaccines, those who get their information from social media, and younger people are particularly hesitant to vaccinate. However, despite this “vaccine hesitancy,” the COVID-19 vaccination rate in Japan is higher than in other countries.

The Vaccine Confidence Index in Japan is known to be one of the lowest in the world. ^34^ One reason for this is that the MHLW, which promotes immunization, itself had concerns about the safety of the HPV vaccine and stopped actively recommending it in 2013. ^35^ In response to this, Japan’s Ministry of Health and Welfare did what many scientists consider to be a terrible mistake. Some even commented that they were pushed by anti-vaccine activists, who claimed that there were side effects, to stop recommending vaccination to prevent cervical cancer.” ^36^

Japan initially approved GlaxoSmithKline’s HPV bivalent vaccine (which protects against the two HPVs with the highest cancer risk) in 2009 and Merck’s quadrivalent vaccine in 2011; in April 2013, the MHLW added both to the national immunization program and began recommending vaccination. However, just 10 weeks later, a number of girls complained of chronic pain, headaches, and motor problems after being vaccinated. The advisory committee therefore proposed to discontinue the recommendation, and the MHLW temporarily suspended the active recommendation in June 2013. As a result, the HPV vaccination coverage plummeted from about 70% to less than 1%, and then to 0.1%. ^37^

However, in November 2021, the MHLW announced that it would begin recommending HPV vaccinations in April 2022, on the grounds that the safety of the vaccine had been confirmed. The reason for this is that the HPV vaccination in Japan has been confirmed to be safe. This was based on a campaign to call for vaccination in Japan, as well as academic studies that showed that such safety issues did not appear in clinical trials. ^38^ Another major factor was the announcement by the WHO’s Global Advisory Committee on Vaccine Safety in 2017 that, after an extensive review of studies from around the world, the vaccine was “extremely safe.” ^39^

The Advisory Committee of the MHLW decided that there was no reason not to resume recommending HPV vaccination. ^40^ In addition, in conjunction with this, the MHLW issued a catch-up vaccination guide in March 2022, for those who missed the HPV vaccination. This refers to a measure that allows women with birthdays between April 2, 1997 and April 1, 2006 who did not receive the HPV vaccine when they should have received it by public expense. Normally, the age range for routine HPV vaccination is from the sixth grade of elementary school to the first grade of high school, that means 12–16 years old. However, as evidenced by the extremely low vaccination rate, many people miss out on HPV vaccination during this period. Therefore, the MHLW decided to offer a new HPV vaccination opportunity for those who are over 16 years old who have not yet been vaccinated. The MHLW has set that period as three years, from April 2022 to March 2025. ^41^ The procedure for receiving the catch-up vaccination is that each eligible person will receive a notice from the municipality in which she has a certificate of residence, which she will take to a medical institution for the vaccination.

The MHLW has a “Q and A” section on the HPV vaccine on its website. One of the questions is, “Why is there a new vaccination opportunity for women born between 1997 and 2005? The answer to this question is: “Because we are going to offer the opportunity for vaccination to women born between 1997 and 2005. In answer, it says, “From 2013 to 2021, efforts to recommend individual HPV vaccination were withheld. The reason for this, it says, was that “there was a situation in which it was not possible to provide sufficient information on the various symptoms, that could occur after vaccination. It then states that at a meeting of experts in November 2021, it was confirmed that there were no particular concerns about safety and that the effectiveness of vaccination clearly outweighed the risk of adverse reactions, and thus the HPV vaccine was recommended.

## Social structure of vaccine hesitation

The web version of the Yomiuri Shimbun, a national newspaper, introduced the experience of one late teenage college student, A, who became ill after receiving the HPV vaccine. ^42^ A received the first HPV vaccine at the end of her second year of junior high school, and received the A received the first HPV vaccine at the end of her eighth-grade year, and the second in June of her junior year. Two days later, a lump about 3 cm in diameter appeared where she had been vaccinated, and it became painful. When she went to the clinic where she had received the vaccine, the doctor told her that it was a common occurrence. A’s mother was concerned and did some research on the Internet, and found that girls who had been vaccinated against HPV had similar symptoms. Then, she contacted the Association for Victims.

After that, A and A’s mother went to the clinic where the vaccine was administered to have a medical certificate written that it was an adverse reaction to the HPV vaccine. However, the doctor at that clinic did not listen to A very much and did not write a diagnosis, saying that he did not think the HPV vaccine was the cause. After that, A visited many hospitals and clinics, but her health showed no sign of getting better.

During spring break just before her sophomore year of high school, A went to a reputable osteopathic clinic where many people who had become physically ill after vaccination went. When she told the practitioner at the clinic about her history and symptoms, he listened carefully to her story. This practitioner also palpated the patient and said that it might be an adverse reaction to the vaccine, and told her not to worry because she would recover.

These words made A feel relieved and happy. She had never had anyone tell her that she would be cured, but this practitioner assured her that she would be cured and did not deny that it was a vaccine side reaction. The practitioner listened carefully to A and A’s mother’s story and said, “I’m sorry you had a hard time.” A’s mother believes that the practitioner expressed hope that she would recover, and that the exercise and diet regimens were effective.

Many healthcare professionals have an attitude of suspicion of “fraud,” refusing to listen to the voices of those who have suffered health problems after receiving vaccines because of the low risk of side effects of vaccines and the scientifically proven fact that vaccines are “extremely safe.” However, this attitude of health professionals creates distrust from the people concerned and society.

In addition, the author wrote earlier that an expert meeting on the reapproval of the HPV vaccine was held in November 2021. Prior to this meeting, on August 30, 2021, a letter was sent to the Prime Minister, the Chief Cabinet Secretary, and the Minister of Health, Labor and Welfare from the “Diet Members Caucus for the Active Resumption of HPV Vaccine Appreciation.” ^43^ A letter of request was entitled “Request for Prompt Resumption of Active Recommendation of HPV Vaccine.” It stated that since the manufacturers of the HPV vaccine had prepared for a considerable number of inoculations to be available, “it is possible that a situation may arise in which the vaccine that has been prepared may have to be discarded due to expiration of its use”.

With this, members of the Ombudsperson Conference on Drug Harms have criticized the Diet members for requesting the MHLW to recommend the vaccine in order to contribute to the HPV vaccine manufacturers. Furthermore, the Expert Council of the MHLW has also issued an opinion accusing the MHLW of following the Diet members’ request and deciding to start catch-up vaccinations. ^44^ This also arouses people’s concerns about the government/administration and experts who recommend vaccination. It is possible that people’s “vaccine hesitancy” is caused by the medical profession’s cold attitude toward vaccine victims and distrust of the government and administration, which seem to give priority to the interests of vaccine manufacturers.

## Beyond the vaccine hesitancy problem

As we have seen above, the problem of “vaccine hesitancy” is accompanied by reasons that differ from scientific evidence. Therefore, the “vaccine hesitancy” problem is not something that can be resolved by improving people’s health literacy and learning about vaccines correctly, but can be seen as a problem that transcends science. Alvin Weinberg, a nuclear physicist, used the term “trans-science” to indicate that there are social issues that science can ask, but science cannot answer. He wrote as following; “Many of the issues which arise in the course of the interaction between science or technology and society— *e.g.*, the deleterious side effects of technology, or the attempts to deal with social problems through the procedures of science—hang on the answers to questions which can be asked of science and *yet which cannot be answered by science.*” ^45^

There are countless questions in medicine that cannot be asked by science. American physician and author Lewis Thomas wrote this about American medicine and society. According to Thomas, the only scientific truth of which we can be entirely confident is that we are utterly ignorant of nature. The sudden confrontation with the depth and scope of “ignorance” is the most important contribution that 20th century science has made to human intelligence. ^46^ Thomas demonstrates the importance of being aware that one cannot be scientifically correct. Regarding the difficulty of judging some things even as an expert, David Bazelon, a judge and jurist, puts it this way. He wrote that in judging factual issues, such as the magnitude of the risks arising from an activity, we as a society should rely on those with the appropriate expertise. However, even in this formulation, many problems remain. There is no clear line between issues of values and issues of fact, and even when properly positioned as issues of scientific fact, there is often no consensus or certainty, even among scientists. Many problems of scientific speculation are in the realm of “trans-science” and cannot be resolved by scientific method and experimentation. ^47^ Medical sociologist Renée Fox has shown in her classic work that medicine is with “uncertainty.” ^48^ Uncertainty has been central to her work since the beginning of medical sociology. Fox then demonstrated the need for careful consideration of the treatment and mishandling of uncertainty and the resulting biohazards, medical risks, and adverse health effects that can result.

It can be said that the issue of “vaccine hesitancy” is truly a “trans-scientific” issue, and it involves “uncertainty”. How can we approach such a problem that transcends science? It is important for both the medical profession and the government to recognize that not everything is clear, that there are areas of “ignorance,” and that there is “uncertainty” when dealing with vaccines. It is sometimes pointed out that behind the “vaccine hesitancy” is a movement of anti-intellectualism and anti-expertism that has no scientific basis. However, simply viewing “vaccine hesitancy” as “anti-intellectualism” and criticizing it is not the answer to the problem. The reason for “vaccine hesitancy” is not so much a distrust of vaccines *per se*, but rather a distrust of the regimes that are promoting vaccines: medical, political, and social entities. What can be done to eliminate this distrust? For example, to understand the pain of those who complain of health problems after receiving the vaccines and respond to them with sincerity is necessary. And not call those women who have suffered health problems by vaccination liars, saying that it is not the vaccine’s fault or that they are fraudulent. It is important for experts, governments, and administrations to take the “third way” by accepting the pain of women who have suffered health problems and then advocating for the prevention of cervical cancer. This is true for any vaccine.

Kentaro Iwata, a Japanese physician, wrote that “Zero risk is just an impossible illusion”. ^49^ He continued, “It is important to supply vaccines that are safe to some extent and that make more sense than the immediate misery”. In Japan, 3,000 people die each year from cervical cancer, not only because of the low vaccination rate, but also because of the low screening uptake rate. This is also sincerely acknowledged, and the need to consider disease prevention not only with vaccines but also with medical checkups is pointed out. When people’s trust in the medical profession, government, and administration is low, no matter how much the government recommends vaccination, the public will not trust vaccines. In order to solve the problem of “vaccine hesitancy”, it will be essential to build people’s trust in the medical profession, politics, and government, even if this may seem like a roundabout way.

## Data availability

### Underlying data

There are no underlying data associated with this article.
