# Supplementary material for: Vaccine Hesitancy in Japan: From a Perspective on Medical Uncertainty and Trans-Scientific Theory
Source: F1000Res. 2023 Jan 11;11:1103. Originally published 2022 Sep 27. [Version 2] doi: 10.12688/f1000research.125159.2 (PMC9849825; doi:10.12688/f1000research.125159.2)
Supplement: Peer review history

# Peer review: Reviewer response for version 2

**DOI:** 10.5256/f1000research.142317.r159847

**Published:** 2023-01-18

**Authors:** Carrie Mae Long

**Affiliations:** 1 Laboratory of Bacteriology, Division of Intramural Research, National Institute of Allergy and Infectious Diseases, National Institutes of Health, Hamilton, MT, USA

The authors revisions have addressed my concerns regarding the initial submission. I believe that this manuscript is suitable for indexing.

Is the topic of the opinion article discussed accurately in the context of the current literature?

Partly

Are arguments sufficiently supported by evidence from the published literature?

Partly

Are all factual statements correct and adequately supported by citations?

Partly

Are the conclusions drawn balanced and justified on the basis of the presented arguments?

Partly

Reviewer Expertise:

Immunology, vaccinology, bacteriology.

I confirm that I have read this submission and believe that I have an appropriate level of expertise to confirm that it is of an acceptable scientific standard.

# Peer review: Reviewer response for version 1

**DOI:** 10.5256/f1000research.137437.r153930

**Published:** 2022-12-13

**Authors:** Tomoko Steen

**Affiliations:** 1 Department of Microbiology and Immunology, School of Medicine, Georgetown University, Washington, DC, USA

**General Comment:**

 Professor Hosoda has done substantial research on the topic through a literature search on relevant articles and websites. Many of the statements here are backed up with appropriate references, but some are missing. I discuss them below.

 It is shocking that Japan, a homogenous country with obedient populations, is facing substantial vaccine hesitation. The proposal to improve Japanese people’s health literacy, explaining the link between “public health and vaccine,” as well as improving communication between medical professionals and the public, might be one of the only solutions to reduce vaccine hesitancy in Japan. Yet, this is a complex issue globally. It is not only communication between the public and health care providers or public health literacy. Much of this is a political belief held by some individuals. I argue this because many healthcare providers are hesitating to take vaccines.

 Vaccine hesitancy is a serious public health concern and a complex issue to solve. The situation is more significant in the United States, where political issues participate in public health decisions. Until I read Professor Hosoda’s article, I was not fully aware of the extent of vaccine hesitancies in Japan. However, on one occasion, I was shocked to hear that a Japanese colleague (a daughter of a leading medical school professor in Japan) said that they hesitated to obtain the vaccine because of a severe side effect reported. If the vaccine’s side effects happen to you or your loved ones, it is 100% risk; an individualistic country such as the United States would be the first to refuse vaccines if higher stakes are reported while your mission is to take a vaccine for the common good.

 Vaccine hesitancy should not be a political issue, but it is in many countries. Overall, Professor Hosoda’s analysis is highly informative, and the article should be indexed after minor revisions.

 Professor Hosoda’s substantial case studies are quite impressive. I recommend indexing after a minor proof read, and revisions. 

**Specific Comments:**

 Some critical information or logical argument needs to be included in some areas.

1. Page 1 For example, in the “Abstract,” *“Therefore, it is said that how to increase the vaccination rate of the population is a major public health challenge. Hence, the substantial number of people who do not receive vaccinations due to "vaccine hesitancy" is problematic.”* This is a confusing sentence: The author needs to explain “herd immunity” before these sentences, then the section after these sentences makes sense.
2. Page 1 I also do not necessarily fully agree with the author’s conclusion here: *“By using medical uncertainty and trans-scientific theory, this paper argues that the problem of "vaccine hesitancy" can be reduced if medical professionals and governments show sincere empathy and attitude toward victims of adverse vaccine reactions and those who hesitate to vaccinate.”* I think education/health literacy and “mutual aid”/familiar good spirit are more important than promoting empathy in one group of people.
3. Page 3 The author needs to add a citation here: *“According to The Japan Society of Gynecologic Oncology, the HPV vaccination rate was as high as approximately 70% when this system was first introduced.”*
4. Page 3 The author did not mean “girls” but rather “symptoms of girls” or “side effects girls experience” in the sentence here. *“The mass media repeatedly reported these girls on TV and in newspapers as side effects of the vaccine.”*
5. Page 3 You meant “the side effects of HPV (missing the word “vaccine”)” here. *“In addition, a great deal of information about the side effects of HPV was circulated on social networking services, further increasing the public’s distrust of the vaccine.”*
6. Page 3 “This is the concept of ‘herd immunity,’ which is a basic public health mechanism” No definition of proper “herd immunity” is in the article; I suggest adding the following:
  A proper definition of Herd Immunity:
  - Herd immunity is reached when a sufficient proportion of the community in a defined area develops a threshold level of immunity to new infection that prevents exponential spread of infections in that community”. For COVID-19, the effective reproduction number [R0], the number of community members infected by each case of virus in question, has been used as reference. This number has to be below one to avoid spread ^1^^-^^4^. 
7. Page 4: Besides reference 10 of Tokyo Shinbun, the author should check the governmental information at the COVID-19 site on this issue should cite. 

Is the topic of the opinion article discussed accurately in the context of the current literature?

Yes

Are arguments sufficiently supported by evidence from the published literature?

Yes

Are all factual statements correct and adequately supported by citations?

Yes

Are the conclusions drawn balanced and justified on the basis of the presented arguments?

Yes

Reviewer Expertise:

Population genetics, epidemiology, microbiome, immune system, gut-brain axes, biomedical science policy and advocacy, and clinical pharmacology.

I confirm that I have read this submission and believe that I have an appropriate level of expertise to confirm that it is of an acceptable scientific standard, however I have significant reservations, as outlined above.

## References

[1] : What Is Herd Immunity?. *JAMA* .2020;324(20) : 10.1001/jama.2020.20895 10.1001/jama.2020.20895 33074287

[2] : Herd Immunity: Understanding COVID-19. *Immunity* .2020;52(5) : 10.1016/j.immuni.2020.04.012 737-741 10.1016/j.immuni.2020.04.012 32433946PMC7236739

[3] : Complexity of the Basic Reproduction Number (R0). *Emerg Infect Dis* .2019;25(1) : 10.3201/eid2501.171901 1-4 10.3201/eid2501.171901 30560777PMC6302597

[4] : Rethinking Herd Immunity: Managingthe Covid-19 Pandemic in a DynamicBiological and Behavioral Environment. *NEJM Catalyst* .2021; Reference source

# Response

**Published:** 2022-12-16

**Authors:** Miwako Hosoda

**Affiliations:** Seisa University, Japan

Dear Prof. Tomoko Steen

 Thank you very much for your review. I learned a lot from your suggestion. I would like to answer each of your comments.

**Specific Comments:**

 1.Page 1 For example, in the “Abstract,” *“Therefore, it is said that how to increase the vaccination rate of the population is a major public health challenge. Hence, the substantial number of people who do not receive vaccinations due to "vaccine hesitancy" is problematic.”* This is a confusing sentence: The author needs to explain “herd immunity” before these sentences, then the section after these sentences makes sense.

**Response 1**

 I added the following explanation in the earlier point of the paper with the references.

 "Herd immunity means that the risk of person-to-person transmission is reduced when a significant portion of the population becomes immune to an infectious disease. While it is not appropriate to overemphasize the benefits of herd immunity, it is certainly possible to confirm its substantial effectiveness."

 Anderson R.M., May R.M. Vaccination and herd immunity to infectious diseases. *Nature. *1985;318:323–329.

 Randolph HE, Barreiro LB. Herd Immunity: Understanding COVID-19. Immunity. 2020 May 19;52(5):737-741. doi: 10.1016/j.immuni.2020.04.012. PMID: 32433946; PMCID:

 2.Page 1 I also do not necessarily fully agree with the author’s conclusion here: *“By using medical uncertainty and trans-scientific theory, this paper argues that the problem of "vaccine hesitancy" can be reduced if medical professionals and governments show sincere empathy and attitude toward victims of adverse vaccine reactions and those who hesitate to vaccinate.”* I think education/health literacy and “mutual aid”/familiar good spirit are more important than promoting empathy in one group of people.

**Response 2**

 Thank you for pointing this out. For the sake of accuracy, I have made the following changes.

 "Various approaches have been pointed out to reduce vaccine hesitancy, including education, health literacy, and consideration about individual stress level."

 Biasio LR. Vaccine hesitancy and health literacy. Hum Vaccin Immunother. 2017 Mar 4;13(3):701-702. doi: 10.1080/21645515.2016.1243633. Epub 2016 Nov 3. PMID: 27808587; PMCID: PMC5360145.

 Zhang H, Li Y, Peng S, Jiang Y, Jin H, Zhang F. The effect of health literacy on COVID-19 vaccine hesitancy among community population in China: The moderating role of stress. Vaccine. 2022 Jul 30;40(32):4473-4478. doi: 10.1016/j.vaccine.2022.06.015. Epub 2022 Jun 8. PMID: 35710509; PMCID: PMC9174466.

 3. Page 3 The author needs to add a citation here: *“According to The Japan Society of Gynecologic Oncology, the HPV vaccination rate was as high as approximately 70% when this system was first introduced.”*

**Response 3**

 I added the following papers as reference.

 Sekine M, Kudo R, Yamaguchi M, Hanley SJB, Hara M, Adachi S, Ueda Y, Miyagi E, Ikeda S, Yagi A, Enomoto T. Japan's Ongoing Crisis on HPV Vaccination. Vaccines (Basel). 2020 Jul 6;8(3):362. doi: 10.3390/vaccines8030362. PMID: 32640691; PMCID: PMC7565470.

 Kudo R, Yamaguchi M, Sekine M, Adachi S, Ueda Y, Miyagi E, Hara M, Hanley SJB, Enomoto T. Bivalent Human Papillomavirus Vaccine Effectiveness in a Japanese Population: High Vaccine-Type-Specific Effectiveness and Evidence of Cross-Protection. J Infect Dis. 2019 Jan 9;219(3):382-390. doi: 10.1093/infdis/jiy516. PMID: 30299519; PMCID: PMC6325350. 

 4. Page 3 The author did not mean “girls” but rather “symptoms of girls” or “side effects girls experience” in the sentence here. *“The mass media repeatedly reported these girls on TV and in newspapers as side effects of the vaccine.”*

**Response 4**

 I changed as follows. 

 “The mass media repeatedly reported these symptoms of girls on TV and in newspapers as side effects of the vaccine.”

 5. Page 3 You meant “the side effects of HPV (missing the word “vaccine”)” here. *“In addition, a great deal of information about the side effects of HPV was circulated on social networking services, further increasing the public’s distrust of the vaccine.”*

**Response 5**

 I added "vaccine" after HPV.

 “In addition, a great deal of information about the side effects of HPV vaccine was circulated on social networking services, further increasing the public’s distrust of the vaccine.”

 6. Page 3 “This is the concept of ‘herd immunity,’ which is a basic public health mechanism” No definition of proper “herd immunity” is in the article; I suggest adding the following:

 A proper definition of Herd Immunity:

 - Herd immunity is reached when a sufficient proportion of the community in a defined area develops a threshold level of immunity to new infection that prevents exponential spread of infections in that community”. For COVID-19, the effective reproduction number [R0], the number of community members infected by each case of virus in question, has been used as reference. This number has to be below one to avoid spread ^1^ ^-^ ^4^ . 

**Response 6**

 I added the explanation of "herd immunity" by following the advice.

 "Herd immunity is reached when a sufficient proportion of the community in a defined area develops a threshold level of immunity to new infection that prevents exponential spread of infections in that community”. For COVID-19, the effective reproduction number [R0], the number of community members infected by each case of virus in question, has been used as reference. This number has to be below one to avoid spread ^1^ ^-^ ^4^ ."

**References**

 1. Desai A, Majumder M: What Is Herd Immunity?. *JAMA*. 2020; **324** (20). Publisher Full Text

 2. Randolph HE, Barreiro LB: Herd Immunity: Understanding COVID-19. *Immunity*. 2020; **52** (5): 737-741 PubMed Abstract | Publisher Full Text

 3. Delamater PL, Street EJ, Leslie TF, Yang YT, et al.: Complexity of the Basic Reproduction Number (R0). *Emerg Infect Dis*. 2019; **25** (1): 1-4 PubMed Abstract | Publisher Full Text

 4. Barker P, Hartley D, Beck AF, Oliver GH, et al.: Rethinking Herd Immunity: Managingthe Covid-19 Pandemic in a DynamicBiological and Behavioral Environment. *NEJM Catalyst*. 2021. Reference Source 

 7. Page 4: Besides reference 10 of Tokyo Shinbun, the author should check the governmental information at the COVID-19 site on this issue should cite. 

**Response 7.**

 I added a report of MHLW which shows a mechanism of the side effect victims. The exact number of the people who got side effect was only announced to the mass media, so I keep putting the reference of Tokyo shinbun.

 Ministry of Health, Labour and Welfare, Health Risk Relief for Novel Coronavirus Vaccine Infectious Disease and Immunization Review Subcommittee Meeting December 9, 2021, 145th Disease and Disability Certification Examination Committee, December 9, 2021. (Japanese)

 Thank you again for your careful review. If you have any questions and/or concerns, don't hesitate to contact with me.

 Best regards,

 Miwako Hosoda

# Peer review: Reviewer response for version 1

**DOI:** 10.5256/f1000research.137437.r151669

**Published:** 2022-11-22

**Authors:** Nelson Filice de Barros

**Affiliations:** 1 Faculdade de Ciências Médicas, Departamento de Saúde Coletiva, Universidade Estadual de Campinas, Campinas, Brazil

Professor Hosoda’s paper reviews studies on "vaccine hesitancy" in Japan, discuss this issue with trans-scientific theory, and points that the "vaccine hesitancy" can be reduced if medical professionals and governments show sincere empathy and attitude toward victims of adverse vaccine reactions and those who hesitate to vaccinate.

 As Professor Hosoda affirms, “the reason for “vaccine hesitancy“ is not so much a distrust of vaccines per se, but rather a distrust of the regimes that are promoting vaccines: medical, political, and social entities”. They present a very important, contemporary, event related to the HPV vaccination in Japan and its relationship with “vaccine hesitancy” in Japan and other places. To Professor Hosoda, the hesitations about vaccines are related to: negative “rumors” about side effects and vaccination itself, risk perception toward vaccines and burden to vaccinate.

 Resistance to vaccination has been present in different countries since the second half of XIX Century ^1^^-^^3^ and, certainly, it is related to the COVID-19 anti-vaccination movement. However, to better understand the “vaccine hesitance” related to the COVID-19 pandemic Professor Hosoda could introduce in their paper the recent debate about the intellectual property of the COVID-19 vaccines and the "apartheid of vaccines".

 The “vaccine hesitancy”, for Professor Hosoda, is co-related it with trans-scientific theory. They assume, as Alvin Weinberg, that there are “questions which can be asked of science and yet which cannot be answered by science”. However, it could be positive to expand the trans-scientific theory with the biopolitics perspective, from Michel Foucault. ^4^^,^^5^ The hesitations about vaccines are less related to the science issues and more associated to the political issues, because the “the quest for certainty in science is ongoing” ^6^, but many times the science consensus, by definition very different from truth, has been used as unquestionable truth, and serving as social technology of control.

 Professor Hosoda finalises the paper affirming that “it will be essential to build people’s trust in the medical profession, politics, and government”. Their position is very close to the idea of the “century of the patient” ^7^, which states that “the health care system inherited from the 20th century falls short on doctors and patients. (…) The 21st century should become the century of the patient (…) centered around patients—not industries, organizations, or doctors”. Considering the proximity of their ideas it could be important whether they discuss some of the seven “sins” the authors developed.

 Although I have suggested expanding the discussion with "apartheid of vaccines" and biopolitics, this does not mean that the article is superficial in relation to the debate on "vaccine hesitancy", on the contrary, it is a very representative text on the issue of vaccination in Japan.

Is the topic of the opinion article discussed accurately in the context of the current literature?

Yes

Are arguments sufficiently supported by evidence from the published literature?

Yes

Are all factual statements correct and adequately supported by citations?

Partly

Are the conclusions drawn balanced and justified on the basis of the presented arguments?

Yes

Reviewer Expertise:

Sociology of Health and Public Health

I confirm that I have read this submission and believe that I have an appropriate level of expertise to confirm that it is of an acceptable scientific standard.

## References

[1] : Priority, invisibility and eradication: the history of smallpox and the Brazilian public health agenda. *Med Hist* .2009;53(2) : 10.1017/s002572730000020x 229-52 10.1017/s002572730000020x 19367347PMC2668879

[2] : Identifying and addressing vaccine hesitancy. *Pediatr Ann* .2015;44(4) : 10.3928/00904481-20150410-07 e71-5 10.3928/00904481-20150410-07 25875982PMC4475845

[3] Parents eye Austrian asylum in Italy vaccination dispute. *BBC News* .2017; Reference source

[4] : The Biopolitics of Social Distancing. *Soc Media Soc* .2020;6(3) : 10.1177/2056305120947661 2056305120947661 10.1177/2056305120947661 34192024PMC7417960

[5] : The competing meanings of "biopolitics" in political science. Biological and postmodern approaches to politics. *Politics Life Sci* .31(1-2) : 10.2990/31_1-2_2 2-15 10.2990/31_1-2_2 23379312

[6] : Addressing vaccination hesitancy in Europe: a case study in state–society relations. *European Journal of Public Health* .2018;28(suppl_3) : 10.1093/eurpub/cky155 30-33 10.1093/eurpub/cky155 PMC620981530383256

[7] : Launching the Centuryof the Patient, In: Better Doctors, Better Patients, Better Decisions: Envisioning Health Care 2020,” edited by G. Gigerenzerand J.A.M. Gray. *Strüngmann Forum Report, vol. 6, J. Lupp, series ed. Cambridge, MA: MIT Press* .2020; Reference source

# Response

**Published:** 2022-12-16

**Authors:** Miwako Hosoda

**Affiliations:** Seisa University, Japan

Dear Dr. Nelson Filice de Barros,

 Thank you very much for your review. I learned a lot from your comments. I update the current version of manuscript by responding the other reviewers' comments. Thank you again for your contribution.

 Best regards,

 Miwako

# Peer review: Reviewer response for version 1

**DOI:** 10.5256/f1000research.137437.r151664

**Published:** 2022-10-11

**Authors:** Carrie Mae Long

**Affiliations:** 1 Laboratory of Bacteriology, Division of Intramural Research, National Institute of Allergy and Infectious Diseases, National Institutes of Health, Hamilton, MT, USA

The submitted manuscript aims to describe vaccine hesitancy in Japan and provide recommendations for the reduction of this phenomenon based on the concepts of “medical uncertainty” and “trans-scientific theory”. The author suggests that empathetic attitudes shown by governmental and medical professionals are a key consideration in addressing vaccine hesitancy. The manuscript addresses an important, timely topic and provides valuable insight. Indeed, a growing body of literature addressing this theme exist; however, I feel that the submitted manuscript does not adequately capture this breadth. Furthermore, the author’s arguments were not consistently supported by the published literature. I feel that the manuscript’s clarity and organization could be improved. Major and minor comments are as follows:

**Major Comments:**

- I felt that the manuscript could be more concisely written, allowing for the inclusion of more prescient information. This was especially apparent in the abstract, which I feel could be shortened and more focused. Further, the general organization of the manuscript could potentially be improved by renaming select section headers (e.g. “Overview of immunization in Japan” to “Overview of vaccine hesitancy in Japan”; There are two sections named “What is vaccine hesitancy?”).
- At times, the author’s statements were not supported by citations. For example: “It is estimated that 5-10% of the vaccine hesitant population has strong antipathy…”; “To preemptively conclude, “vaccine hesitancy” is not caused by distrust of vaccines or the risk of vaccine side effects, as is generally believed, but rather by distrust of the medical…”. Further, the author states that “no matter how much the medical profession, government, and administration scientifically prove the safety of vaccines, and no matter how much they claim that people’s health damage is not caused solely by vaccine side effects, the mindset of those who are “vaccine hesitant” will not change.” There are numerous studies that suggest the opposite ^1^^-^^4^. In addition, there are studies addressing the author’s core argument of the importance of empathy and I feel that these studies should be cited ^5^^-^^9^. Overall, I feel that amelioration of the lack of relevant citations and ensuing discussion would strengthen the author’s arguments and the manuscript as a whole.
- I feel that the concepts of “medical uncertainty” and “trans-scientific theory” should be defined earlier in the manuscript and described more comprehensively, given their importance to the author’s central argument. 

**Minor Comments:**

- I felt that the use of quotation marks was not consistent. Perhaps this could be addressed for clarity.
- The statement that “People with underlying diseases or immune disorders, such as allergies, are considered to by at high risk from vaccinations.” is not necessarily true for all vaccines. I suggest clarifying this statement as most contraindications are vaccine/platform specific.
- Is the “MR” vaccine reference in the “Vaccine hesitancy 3): burden to vaccinate” section referring to the “MMR” vaccine?

Is the topic of the opinion article discussed accurately in the context of the current literature?

Partly

Are arguments sufficiently supported by evidence from the published literature?

Partly

Are all factual statements correct and adequately supported by citations?

Partly

Are the conclusions drawn balanced and justified on the basis of the presented arguments?

Partly

Reviewer Expertise:

Immunology, vaccinology, bacteriology.

I confirm that I have read this submission and believe that I have an appropriate level of expertise to state that I do not consider it to be of an acceptable scientific standard, for reasons outlined above.

## References

[1] : Addressing vaccine hesitancy: Clinical guidance for primary care physicians working with parents. *Can Fam Physician* .65(3) :175-181 30867173PMC6515949

[2] : Countering vaccine hesitancy through medical expert endorsement. *Vaccine* .2022;40(32) : 10.1016/j.vaccine.2022.06.031 4635-4643 10.1016/j.vaccine.2022.06.031 PMC921708435750542

[3] : Countering vaccine hesitancy through immunization information systems, a narrative review. *Hum Vaccin Immunother* .2019;15(11) : 10.1080/21645515.2019.1599675 2508-2526 10.1080/21645515.2019.1599675 30932725PMC6930057

[4] : COVID-19 vaccination in the UK: Addressing vaccine hesitancy. *Lancet Reg Health Eur* .2021;1: 10.1016/j.lanepe.2020.100016 100016 10.1016/j.lanepe.2020.100016 34173622PMC7834442

[5] : COVID-19 vaccine hesitancy and media channel use in Japan: could media campaigns be a possible solution?. *Lancet Reg Health West Pac* .2022;18: 10.1016/j.lanwpc.2021.100357 100357 10.1016/j.lanwpc.2021.100357 35005668PMC8720144

[6] : Empathy in vaccination counselling: a survey on the impact of a three-day residential course. *Hum Vaccin Immunother* .2019;15(3) : 10.1080/21645515.2018.1536587 631-636 10.1080/21645515.2018.1536587 30325260PMC6605730

[7] : Vaccine hesitancy and health care providers: Using the preferred cognitive styles and decision- making model and empathy tool to make progress. *Vaccine X* .2022;11: 10.1016/j.jvacx.2022.100174 100174 10.1016/j.jvacx.2022.100174 35782719PMC9241108

[8] : Can Altruistic Emotions Promote Vaccine Advocacy? Examining the Use of Empathy and Elevation in Vaccine Messages. *Journal of Health Communication* .2021;26(11) : 10.1080/10810730.2021.2002981 753-763 10.1080/10810730.2021.2002981 34802392

[9] : COVID-19 Vaccine Hesitancy and Its Associated Factors in Japan. *Vaccines (Basel)* .2021;9(6) : 10.3390/vaccines9060662 10.3390/vaccines9060662 34204465PMC8234307

# Response

**Published:** 2022-12-16

**Authors:** Miwako Hosoda

**Affiliations:** Seisa University, Japan

Dear Dr. Carrie Mae Long,

 Thank you very much for your review. I learned a lot from your suggestion. I would like to answer each of your comments.

**Comment 1:**

- I felt that the manuscript could be more concisely written, allowing for the inclusion of more prescient information. This was especially apparent in the abstract, which I feel could be shortened and more focused. Further, the general organization of the manuscript could potentially be improved by renaming select section headers (e.g. “Overview of immunization in Japan” to “Overview of vaccine hesitancy in Japan”; There are two sections named “What is vaccine hesitancy?”).

**My answer 1:**

 The abstract is within the designated word count, so I think it is fine as it is, but I have shortened it a bit according to your advice.

 I changed the section headers of second “What is vaccine hesitancy?” to "Social Structure that causes vaccine hesitation"

**Comment 2:**

- At times, the author’s statements were not supported by citations. For example: “It is estimated that 5-10% of the vaccine hesitant population has strong antipathy…”; “To preemptively conclude, “vaccine hesitancy” is not caused by distrust of vaccines or the risk of vaccine side effects, as is generally believed, but rather by distrust of the medical…”. Further, the author states that “no matter how much the medical profession, government, and administration scientifically prove the safety of vaccines, and no matter how much they claim that people’s health damage is not caused solely by vaccine side effects, the mindset of those who are “vaccine hesitant” will not change.” There are numerous studies that suggest the opposite ^1^ ^-^ ^4^ . In addition, there are studies addressing the author’s core argument of the importance of empathy and I feel that these studies should be cited ^5^ ^-^ ^9^ . Overall, I feel that amelioration of the lack of relevant citations and ensuing discussion would strengthen the author’s arguments and the manuscript as a whole.

**My answer 2:**

 In order to be more accurate in content, the text has been rewritten as follows. And added supportive references.

 “no matter how much the medical profession, government, and administration scientifically prove the safety of vaccines, and no matter how much they claim that people’s health damage is not caused solely by vaccine side effects, it may change the mindset of those who are at some vaccine hesitant, but it cannot reduce it to zero.”

**Comment 3:**

- I feel that the concepts of “medical uncertainty” and “trans-scientific theory” should be defined earlier in the manuscript and described more comprehensively, given their importance to the author’s central argument. 

**My answer 3:**

 I add the concepts of “medical uncertainty” and “trans-scientific theory” earlier in the manuscript and described more comprehensively.

**Comment 4:**

- I felt that the use of quotation marks was not consistent. Perhaps this could be addressed for clarity.

**My answer 4:**

  I have standardized the use of quotation marks.

**Comment 5:**

- The statement that “People with underlying diseases or immune disorders, such as allergies, are considered to by at high risk from vaccinations.” is not necessarily true for all vaccines. I suggest clarifying this statement as most contraindications are vaccine/platform specific.

**My answer 5:**

 I changed the sentence. 

 “People with underlying diseases or immune disorders, such as allergies, are mostly considered to be at high risk from vaccinations.”

**Comment 6:**

- Is the “MR” vaccine reference in the “Vaccine hesitancy 3): burden to vaccinate” section referring to the “MMR” vaccine?

**My answer 6:**

 In many other countries, the MMR vaccine is used, but in Japan, the MMR vaccine is not used; the MR vaccine is used.

 I have added important references.

 If you have any further questions, please let me know. Thank you again for your effort. 

 Best regards,

 Miwako Hosoda
